# PRIDA: cross-domain protein–RNA interaction prediction based on biological language models and conditional domain-adversarial learning

**DOI:** 10.1093/bioinformatics/btag802

**Published:** 2026-10-29

**Authors:** Yangyang Zhou, Jiahao Li, Simin Liu, Zifan Wang, Runhao Liu, Zexuan Zhu, Tiantian Zhu, Jun Zhang

**Affiliations:** School of Artificial Intelligence, Shenzhen University, Shenzhen, Guangdong 518060, China; National Engineering Laboratory for Big Data System Computing Technology, Shenzhen University, Shenzhen, Guangdong 518060, China; School of Artificial Intelligence, Shenzhen University, Shenzhen, Guangdong 518060, China; National Engineering Laboratory for Big Data System Computing Technology, Shenzhen University, Shenzhen, Guangdong 518060, China; School of Artificial Intelligence, Shenzhen University, Shenzhen, Guangdong 518060, China; National Engineering Laboratory for Big Data System Computing Technology, Shenzhen University, Shenzhen, Guangdong 518060, China; School of Artificial Intelligence, Shenzhen University, Shenzhen, Guangdong 518060, China; National Engineering Laboratory for Big Data System Computing Technology, Shenzhen University, Shenzhen, Guangdong 518060, China; School of Artificial Intelligence, Shenzhen University, Shenzhen, Guangdong 518060, China; National Engineering Laboratory for Big Data System Computing Technology, Shenzhen University, Shenzhen, Guangdong 518060, China; School of Artificial Intelligence, Shenzhen University, Shenzhen, Guangdong 518060, China; National Engineering Laboratory for Big Data System Computing Technology, Shenzhen University, Shenzhen, Guangdong 518060, China; School of Artificial Intelligence, Shenzhen University, Shenzhen, Guangdong 518060, China; National Engineering Laboratory for Big Data System Computing Technology, Shenzhen University, Shenzhen, Guangdong 518060, China; School of Artificial Intelligence, Shenzhen University, Shenzhen, Guangdong 518060, China; National Engineering Laboratory for Big Data System Computing Technology, Shenzhen University, Shenzhen, Guangdong 518060, China

## Abstract

**Motivation:**

The interaction between RNA and proteins is a complex process that involves atom-level details and dynamic changes. Accurate prediction of protein–RNA interactions helps researchers to develop protein-targeted small nucleic acid drugs. However, the scarcity of protein–RNA complexes and experimental data limits the ability of existing data-driven supervised learning methods to learn latent patterns, leading to limited performance.

**Results:**

In this study, we propose PRIDA (Protein–RNA Interaction prediction based on Domain-Adversarial learning), a protein–RNA interaction framework that combines bilinear attention with conditional domain-adversarial learning. Powered by pretrained protein and RNA language models, PRIDA improves interaction prediction under protein-level distribution shifts and limited labeled data. By integrating PRIDA with Monte Carlo Tree Search, we apply it to a virtual screening pipeline to generate RNA aptamer candidates for specific target proteins. Experimental results show that PRIDA achieves state-of-the-art performance in cross-domain protein-RNA interaction prediction and provides effective guidance for RNA aptamer virtual screening.

**Availability and implementation:**

The data and source code of the proposed method are available at GitHub (https://github.com/zhouyyang/PRIDA) and Zenodo (https://zenodo.org/records/23644677).

## 1 Introduction

Protein–RNA interactions (PRIs) are fundamental molecular mechanisms that maintain normal cellular functions (Wilkinson *et al.* 2022) and regulate gene expression (Tang *et al.* 2023). For instance, long noncoding RNAs and small nucleolar RNAs have been shown to be critically involved in cancer (Cao *et al.* 2024, Tian *et al.* 2024), neurodegenerative diseases (Patel *et al.* 2023), and immune disorders (Basavappa *et al.* 2019). Accurate identification of PRIs is crucial for elucidating complex biological mechanisms and developing novel therapeutic strategies.

Structure-based experimental techniques, such as X-ray crystallography (Ke and Doudna 2004), can directly resolve the three-dimensional structures of interacting molecules. However, these approaches are expensive, time-consuming, and technically demanding. Traditional biochemical and molecular biology experiments (Keene *et al.* 2006, Licatalosi *et al.* 2008), although capable of detecting PRIs, are resource-intensive and have limited throughput. To overcome these limitations, researchers have proposed computational approaches, including traditional machine learning methods (Lu *et al.* 2013, Suresh *et al.* 2015) and deep learning-based techniques (Wang *et al.* 2021, Li *et al.* 2024). For example, RPITER (Peng *et al.* 2019) combines an improved joint triad feature encoding scheme to capture both sequence and structural information, and integrates convolutional neural networks (CNNs) with stacked autoencoders for feature extraction and interaction prediction. CBIL-VHPLI (Zhang *et al.* 2024b) employs CNN and bidirectional long short-term memory networks with multiscale features, showing promising performance in viral-host protein–lncRNA interaction prediction. SeqMG-RPI (Ma *et al.* 2025) further improves sequence-based PRI prediction by integrating multiscale RNA features with protein graph representations and multiple neural network modules.

Despite remarkable progress in recent deep learning approaches, predicting PRIs under limited-data scenarios remains a significant challenge. Most existing models rely heavily on large-scale annotated interaction datasets and often exhibit substantial performance degradation when transferred to unseen proteins, RNA families, or independent benchmark datasets. This limitation is particularly problematic for emerging applications such as RNA aptamer discovery, where experimentally validated interaction data are scarce, and the distribution of candidate sequences may differ from those represented in current training datasets. Therefore, developing a PRI prediction framework with improved prediction capability under few-shot settings is of considerable practical and biological importance.

Recent advances in biological language models (Ferruz *et al.* 2022, Lin *et al.* 2023, Madani *et al.* 2023, Hayes *et al.* 2025, Liuzhenghao *et al.* 2025, Penić *et al.* 2025) offer new opportunities for addressing the challenge of limited data in PRI prediction. By pretraining on millions of protein and RNA sequences, these models can learn rich representations that capture evolutionary, structural, and functional properties directly from sequence data. Such transferable biological knowledge has been shown to improve performance across diverse downstream tasks (Shen *et al.* 2024; Zhang *et al.* 2024a, 2026; He *et al.* 2026), particularly when labeled data are scarce. Therefore, biological language models provide a promising foundation for learning PRI patterns under few-shot settings.

However, pretrained representations alone may not fully address the generalization issue caused by distribution shifts among existing PRI datasets. Differences in experimental protocols, biological contexts, and data collection strategies often lead to domain-specific biases, limiting the transferability of predictive models. The conditional domain-adversarial learning (Long *et al.* 2018) offers a potential solution by encouraging the model to learn interaction-relevant features that are invariant across datasets while preserving discriminative information for downstream prediction (Bai *et al.* 2023, Li *et al.* 2025). Combining biological language models with conditional domain-adversarial learning provides a principled framework for improving both prediction accuracy and cross-domain generalization in PRI prediction.

To fill these gaps, we propose PRIDA (Protein-RNA Interaction prediction based on Domain-Adversarial learning), a novel PRI prediction framework that integrates biological LMs with conditional domain-adversarial learning. Specifically, we employ large-scale pretrained protein and RNA language models to extract informative sequence representations, and introduce a conditional domain-adversarial strategy to reduce dataset-specific biases and improve the transferability of learned interaction patterns across domains. By jointly learning discriminative and domain-invariant features, PRIDA aims to improve predictive robustness and generalization when only a limited number of training samples are available.

To evaluate the proposed method, we conducted experiments across three PRI benchmark datasets with homology-isolated Source and Target domains. Then we extended PRIDA to analyze protein–aptamer interactions and incorporated it into a virtual aptamer screening pipeline by coupling PRIDA with a Monte Carlo search-based reinforcement learning strategy.

Experimental results demonstrate that PRIDA outperforms existing state-of-the-art methods across three RPI benchmark datasets and protein–aptamer interaction prediction, particularly under cross-domain and limited-data settings. These findings suggest that PRIDA provides a new, robust solution for PRI prediction and can help develop a practical computational tool to facilitate functional RNA discovery and rational RNA therapeutic design.

## 2 Materials and methods

### 2.1 Datasets

We evaluated different methods on RPI369 (Muppirala *et al.* 2011), RPI488 (Pan *et al.* 2016), RPI1807 (Suresh *et al.* 2015), and a protein–aptamer dataset constructed from experimentally resolved PDB complexes (Berman *et al.* 2000). To control cross-domain protein homology, unique protein sequences were linked when either MMseqs2 (Steinegger and Söding 2017) identity was at least 30% with at least 80% coverage for both sequences or Needleman–Wunsch global identity was at least 30%. Connected components of this graph defined indivisible homology groups, and no group was shared by both Source and Target. In summary, all partitions contained neither exact cross-domain duplicates nor qualifying sequence-similarity matches.

For each RPI dataset, five distinct homology-isolated Source/Target partitions were constructed while preserving the Source-training, Target-training, Target-test sizes, and all interaction records, as listed in Table 1. Pairwise Jaccard similarity between the different Target protein sets was constrained to at most 0.40. For the Aptamer task, the fixed Target domain contained 330 interaction pairs, and we pooled the three RPI datasets to build the Source data to broaden protein-family coverage. We then restricted the pooled Source to RNA lengths of at most 126 nt for Aptamer adaptation and removed proteins homologous to any Aptamer Target protein. Finally, we evaluated different methods on the 330 Target pairs using stratified five-fold cross-validation with mutually exclusive 66-pair test folds. Detailed construction, sequence-identity statistics, and distribution visualizations are provided in the Supplementary Material, available as supplementary data at *Bioinformatics* online.

**Table 1:** Sample sizes of the homology-isolated evaluation datasets.^a^

Dataset	Splits	Source train	Target train	Target test	Total
RPI488	5	285	162	41	488
RPI369	5	518	176	44	738
RPI1807	5	1700	1229	308	3237
Aptamer	5	1468	264	66	1798

a Values are identical across the five partitions (or folds) within each dataset. Source train contains labeled samples, target train contains unlabeled adaptation samples, and target test is reserved for evaluation.

### 2.2 Overview of PRIDA

As illustrated in Fig. 1, the framework provides an integrated approach for target-specific cross-domain PRI modeling and RNA aptamer virtual screening. Protein and RNA sequences are first encoded with pretrained biological language models, and their representations are adapted using labeled Source-domain samples. A bilinear attention network explicitly models fine-grained interactions between RNA nucleotides and protein residues. Conditional domain-adversarial learning then uses labeled source-training samples together with unlabeled target-training samples to align joint interaction representations; target-training labels are never exposed, and target-test samples are reserved for evaluation. The resulting predictor is integrated with Monte Carlo tree search (MCTS) to guide *de novo* aptamer sequence search for a protein target of interest.

**Figure 1:**
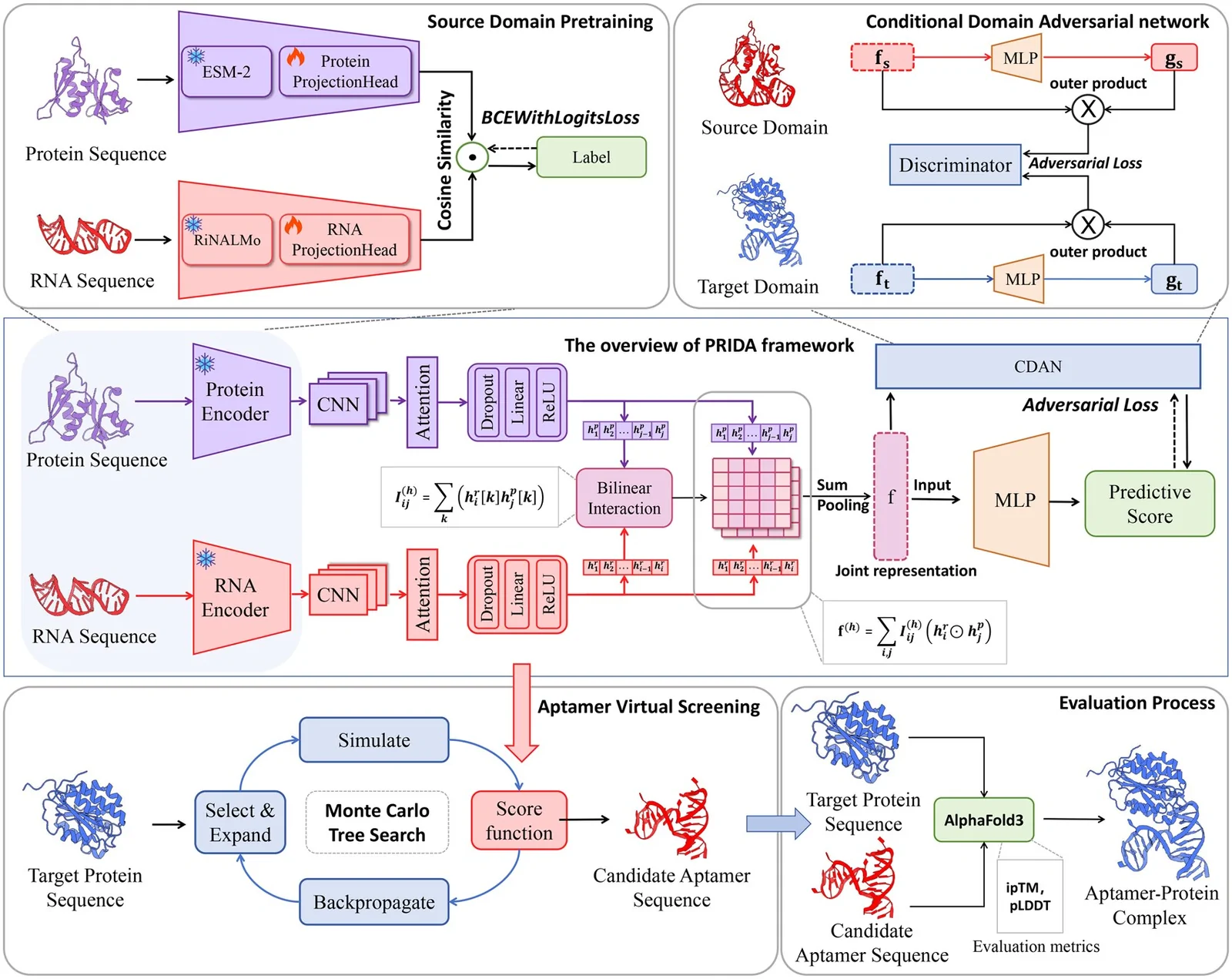
Overview of the proposed cross-domain PRI prediction framework. It first performs source-domain supervised representation alignment for biological language model encoders; then freezes the encoders and combines them with bilinear-attention-network-based interaction modeling and conditional domain-adversarial learning for cross-domain PRI prediction; finally, integrates MCTS for end-to-end nucleic acid aptamer virtual screening.

### 2.3 Source-domain supervised sequence representation learning

Protein and RNA sequences are encoded using the pretrained protein language model ESM-2 (Lin *et al.* 2023) and the RNA language model RiNALMo (Penić *et al.* 2025), respectively. The encoders first produce token-level representation matrices $H_{p}\in R^{B\times L_{p}\times d_{p}}$ and $H_{r}\in R^{B\times L_{r}\times d_{r}}$. Separate projection heads map every token into a shared 512-dimensional space, after which each projected token is L2-normalized. Mask-aware mean pooling over nonpadding tokens yields fixed-length sequence vectors $z_{p},z_{r}\in R^{B\times 512}$.

To improve task-specific discrimination, we introduce a supervised source-domain representation-adaptation stage before cross-domain training. The pretrained encoders remain frozen, and we optimize only the projection heads using labeled source-training PRI data. This stage uses neither target-training samples nor target labels. For a source-domain pair x=(r,p), its interaction tendency is measured by the cosine similarity *s* between $z_{r}$ and $z_{p}$, and the projection heads are optimized by minimizing


(1) $$L_{\text{src}}=-E_{(x,y)∼D_{s}}\left[y\;\text{log}\;\sigma (s)+(1-y)\;\text{log}\;\left(1-\sigma (s)\right)\right],$$

where *y* is the Source-domain interaction label, σ(·) denotes the sigmoid function, and the expectation is approximated by the empirical average over labeled source-training samples.

### 2.4 Feature enhancement module

After obtaining the pretrained sequence embeddings, we employed three-layer CNNs to separately encode the RNA and protein representations. CNN modules capture local sequence patterns and short-range dependencies, which are important for modeling localized binding motifs involved in PRIs. To further highlight informative sequence positions, we introduce an attention mechanism after the CNN encoders. This mechanism adaptively assigns different weights to RNA nucleotides and protein residues, thereby enhancing potentially interaction-relevant features while reducing the influence of redundant or less informative sequence signals.

### 2.5 Bilinear attention network for interaction modeling

To capture fine-grained interaction patterns between RNA and protein sequences, we employ a bilinear attention network (Kim *et al.* 2018) to jointly model their representations. After encoding with a pretrained language model, extracting convolutional features, and enhancing with attention-based features, the RNA and protein sequences are represented at the position level. Let $h_{i}^{r}\in R^{d}$ and $h_{j}^{p}\in R^{d}$ denote the feature vector of the *i*-th RNA nucleotide and the *j*-th protein residue, respectively.

The Bilinear Attention Network explicitly models pairwise interactions between RNA nucleotides and protein residues. For the *h*-th attention head, the bilinear interaction score between the *i*-th RNA nucleotide and the *j*-th protein residue is calculated as follows:


(2) $$e_{ij}^{\left(h\right)}={\left(h_{i}^{r}\right)}^{⊤}W^{\left(h\right)}h_{j}^{p},$$

where $W^{\left(h\right)}\in R^{d\times d}$ is a learnable bilinear parameter matrix for the *h*-th attention head.

The interaction scores are then normalized to obtain the attention weights:


(3) $$\alpha_{ij}^{\left(h\right)}=\frac{\;\text{exp}\;\left(e_{ij}^{\left(h\right)}\right)}{\sum_{i^{\prime }=1}^{L_{r}}\sum_{j^{\prime }=1}^{L_{p}}\;\text{exp}\;\left(e_{i^{\prime }j^{\prime }}^{\left(h\right)}\right)}.$$

Based on these attention weights, protein–RNA joint features are aggregated through weighted element-wise products:


(4) $$f^{\left(h\right)}=\sum_{i=1}^{L_{r}}\sum_{j=1}^{L_{p}}\alpha_{ij}^{\left(h\right)}\left(h_{i}^{r}⊙h_{j}^{p}\right),\;f=\sum_{h=1}^{H}f^{\left(h\right)},$$

where ⊙ denotes element-wise multiplication, $L_{r}$ and $L_{p}$ denote the lengths of the RNA and protein sequences, respectively, and *H* is the number of attention heads.

The resulting joint representation f is subsequently fed into a multilayer perceptron for PRI prediction and is also used for conditional domain-adversarial training in the cross-domain learning framework.

### 2.6 Conditional domain adversarial network

In realistic PRI prediction scenarios, models may encounter target proteins whose distributions differ substantially from those observed during training, degrading performance under protein-level domain shift. To improve cross-domain generalization, we incorporate a conditional domain adversarial network (CDAN) into the proposed framework.

Following the two-stage training strategy, we use pretrained protein and RNA language model encoders as fixed feature extractors during cross-domain training. The encoded representations are then passed to a trainable feature-enhancement and interaction-modeling network, including CNN-based modules, attention mechanisms, and the bilinear attention network, to obtain the joint protein–RNA representation f. Let $\hat{y}=G(f)$ denote the prediction probability vector produced by the interaction predictor G(·).

Unlike conventional domain-adversarial methods that align marginal feature distributions, CDAN conditions domain alignment on the prediction information. Specifically, we construct a conditional representation by combining the joint feature representation with the prediction output:


(5) $$z=f⊗\hat{y},$$

where ⊗ denotes the outer product.

Based on the conditional representation z, a domain discriminator D(·) is trained to distinguish samples from the labeled source domain $D_{s}$ and the unlabeled target domain $D_{t}$. The adversarial loss is defined as follows:


(6) $$L_{\text{adv}}=-E_{x∼D_{s}}\left[\text{log}\;D(z)\right]-E_{x∼D_{t}}\left[\text{log}\;\left(1-D(z)\right)\right].$$

During cross-domain training, the interaction predictor is optimized using labeled source-domain samples, while both source-domain and unlabeled target-domain training samples are used for conditional adversarial alignment. The source-domain classification loss is denoted as $L_{\text{cls}}$. The overall optimization objective follows a minimax formulation:


(7) $$\underset{F,G}{\min}\underset{D}{\max}L_{\text{cls}}(F,G)-\lambda L_{\text{adv}}(F,G,D),$$

where *F* denotes the trainable feature enhancement and interaction modeling network, *G* denotes the interaction predictor, *D* denotes the domain discriminator, and λ controls the trade-off between source-domain prediction accuracy and domain alignment.

Within every partition or fold, source-training labels were used for supervision, target-training labels were not exposed to any model, and CDAN accessed target-training samples only as unlabeled data. Target-test samples were reserved for evaluation.

### 2.7 RNA aptamer virtual screening pipeline

We further integrate the proposed cross-domain PRI prediction model with MCTS to construct a virtual screening framework for generating RNA aptamer candidates. Given a target protein sequence, the framework iteratively searches and evaluates the target RNA sequence within the nucleic acid sequence space to identify candidate aptamer sequences with high predicted interaction potential.

During the search process, MCTS incrementally expands RNA nucleotide sequences to generate candidate aptamers. At each search node, the trained cross-domain PRI predictor evaluates the interaction between the candidate RNA sequence and the target protein. The predicted interaction score serves as a reward signal that guides the search toward regions of sequence space more likely to contain promising aptamer candidates.

By coupling learned interaction evaluation with sequence-level search, the proposed framework provides a computational strategy for recommending target-oriented nucleic acid aptamer candidates under limited labeled data. The Supplementary Material, available as supplementary data at *Bioinformatics* online, provides detailed descriptions of the MCTS search strategy, node expansion rules, and parameter settings.

### 2.8 Evaluation

For PRI prediction, the area under the receiver operating characteristic curve (AUROC) and the area under the precision–recall curve (AUPRC) were the primary ranking endpoints; Accuracy, Precision, F1, and Sensitivity were secondary descriptive metrics calculated at the threshold maximizing F1. Results are reported as the mean ± sample SD over five matched partitions or folds. For AUROC and AUPRC, fold-aligned two-sided one-sample *t*-tests were applied to paired performance differences, followed by Holm–Bonferroni correction within each dataset. Detailed metric definitions and statistical procedures are provided in the Supplementary Material, available as supplementary data at *Bioinformatics* online.

For aptamer generation, the five-fold-specific PRIDA models trained for the Aptamer task jointly provided the MCTS reward by averaging their positive-interaction probabilities. All competing methods generated ten 20-nt candidates for each of 125 unique Target proteins. The common 20-nt length follows recent aptamer-generation precedent (Zhang *et al.* 2023, 2026) and provides a uniform, reproducible, length-matched evaluation setting for all methods. Generated candidates were evaluated using AlphaFold 3 ipTM and pLDDT (Abramson *et al.* 2024) and HDOCK scores (Yan *et al.* 2017). Because molecular-dynamics evaluation of all 125 targets would be computationally prohibitive, 6CF2 and 8TFD were selected a priori for their distinct biological relevance: 6CF2 contains HIV-1 Rev, whose cognate nucleic-acid interaction is central to the viral life cycle (Dearborn *et al.* 2018), whereas 8TFD contains the RNA-binding domain of the SARS-CoV-2 nucleocapsid protein in an aptamer complex (Esler *et al.* 2024). We made this selection independently of the molecular-dynamics results. For both targets, all 10 candidates from every method were subjected to 50-ns molecular-dynamics simulations in GROMACS with the CHARMM36 force field, followed by MM/PBSA binding-free-energy estimation (Denning *et al.* 2011, Miller *et al.* 2012, Abraham *et al.* 2015).

## 3 Results

### 3.1 Baseline methods

To evaluate PRI prediction, we compared PRIDA with five representative baselines: LPI-BLS (Fan and Zhang 2019), CBIL-VHPLI (Zhang *et al.* 2024b), SeqMG-RPI (Ma *et al.* 2025), Transfer-RPI (Zeng *et al.* 2025), and RPI-CapsuleGAN (Wang *et al.* 2023). These methods cover broad learning systems, deep sequence models, graph-enhanced modeling, transfer learning, and generative-adversarial representation learning. RPI-CapsuleGAN is an interaction-prediction baseline, whereas PRIDA specifically performs target-specific adaptation under an explicit protein-level Source–Target shift and subsequently serves as an MCTS reward model.

For aptamer generation, we compared PRIDA-guided MCTS with Apta-MCTS (Lee *et al.* 2021), AptaTrans (Shin *et al.* 2023), and RNA-BAnG (Klypa *et al.* 2025). The first two comparisons isolate the effect of the reward model within MCTS-based generation, whereas RNA-BAnG provides a complementary structure-conditioned reference.

### 3.2 Cross-domain interaction prediction

Table 2 reports the cross-domain results after removal of cross-domain protein homology. Each of the four datasets was treated as a separate target-specific domain-adaptation task. For each dataset, we trained the model using the corresponding Source-training data and unlabeled Target-training data; we reserved the Target-test data for evaluation. We did not share or reuse models across different datasets. PRIDA achieved the highest mean AUROC and AUPRC on all four datasets, leading all comparators in each of the eight dataset–metric combinations defined by these two prespecified primary endpoints. This consistent advantage was especially pronounced on the more difficult RPI369 and RPI1807 partitions, while the fixed-origin Aptamer transfer task remained challenging for all methods.

**Table 2:** Cross-domain interaction-prediction results under protein-level domain shifts using the homology-isolated datasets.^a^

Dataset	Models	AUROC	AUPRC	Accuracy	Precision	F1	Sensitivity
RPI488	PRIDA	**0.931** ± **0.016**	**0.941** ± **0.018**	0.907 ± 0.032	**0.937** ± **0.066**	0.896 ± 0.036	0.863±0.060
	LPI-BLS	0.911±0.064	0.898±0.059***	**0.912** ± **0.033**	0.936 ± 0.041	**0.901** ± **0.043**	0.871±0.088
	SeqMG-RPI	0.887±0.025**	0.875±0.057***	0.902±0.024	0.919±0.068	0.893±0.026	0.863±0.066
	Transfer-RPI	0.926±0.034	0.919 ± 0.030	0.907 ± 0.042	0.927±0.053	0.896±0.041	0.874 ± 0.079
	CBIL-VHPLI	0.902±0.066***	0.912±0.026**	0.902±0.030	0.918±0.061	0.891±0.039	0.865±0.053
	RPI-CapsuleGAN	0.928 ± 0.062	0.919 ± 0.069	0.902±0.069	0.908±0.095	0.895±0.071	**0.884** ± **0.069**
RPI369	PRIDA	**0.597** ± **0.099**	**0.599** ± **0.128**	**0.595** ± **0.089**	**0.572** ± **0.083**	**0.699** ± **0.028**	0.927±0.094
	LPI-BLS	0.340±0.039**	0.407±0.021*	0.509±0.012	0.505±0.007	0.669±0.005	0.991 ± 0.020
	SeqMG-RPI	0.423±0.089*	0.470 ± 0.038 *	0.527±0.049	0.517±0.031	0.676±0.015	0.982±0.041
	Transfer-RPI	0.430 ± 0.119	0.457±0.079*	0.459±0.091	0.448±0.130	0.371±0.099	0.318±0.085
	CBIL-VHPLI	0.255±0.066***	0.378±0.033*	0.514±0.012	0.507±0.006	0.673±0.006	**1.000** ± **0.000**
	RPI-CapsuleGAN	0.313±0.095**	0.394±0.051*	0.536 ± 0.034	0.520 ± 0.020	0.680 ± 0.014	0.982±0.025
RPI1807	PRIDA	**0.726** ± **0.081**	**0.738** ± **0.104**	**0.730** ± **0.054**	**0.726** ± **0.118**	**0.779** ± **0.016**	0.875±0.126
	LPI-BLS	0.472±0.065***	0.512±0.032**	0.584±0.050	0.569±0.035	0.720±0.021	**0.986** ± **0.026**
	SeqMG-RPI	0.575±0.049*	0.617±0.057*	0.569±0.023	0.558±0.014	0.711±0.007	0.981 ± 0.022
	Transfer-RPI	0.669 ± 0.091	0.683±0.101	0.652±0.034	0.660 ± 0.054	0.703±0.028	0.767±0.107
	CBIL-VHPLI	0.620±0.067*	0.694 ± 0.050	0.585±0.066	0.580±0.069	0.715±0.016	0.955±0.098
	RPI-CapsuleGAN	0.655±0.052	0.677±0.077	0.675 ± 0.044	0.650±0.054	0.748 ± 0.020	0.888±0.056
Aptamer	PRIDA	**0.604** ± **0.033**	**0.662** ± **0.050**	0.561 ± 0.034	0.520±0.020	0.681±0.013	0.988 ± 0.027
	LPI-BLS	0.498±0.124**	0.506±0.096*	0.542±0.066	0.527±0.043	0.682 ± 0.025	0.976±0.040
	SeqMG-RPI	0.505±0.035***	0.509±0.029**	0.518±0.025	0.510±0.014	0.674±0.009	**0.994** ± **0.014**
	Transfer-RPI	0.445±0.030***	0.447±0.023***	0.536±0.035	0.535±0.020	0.675±0.033	0.915±0.072
	CBIL-VHPLI	0.584 ± 0.084	0.617 ± 0.086	**0.579** ± **0.091**	**0.567** ± **0.100**	**0.689** ± **0.021**	0.921±0.128
	RPI-CapsuleGAN	0.530±0.068*	0.525±0.046**	0.545±0.085	0.538 ± 0.077	0.679±0.020	0.952±0.108

a Values are mean ± sample SD over five matched partitions or folds. Bold and underlined values denote the best and second-best means within each dataset. For AUROC and AUPRC, asterisks indicate Holm–Bonferroni-adjusted significance relative to PRIDA:

* *P* < 0.05,

** *P* < 0.01, and

*** *P* < 0.001.

PRIDA led all eight prespecified primary dataset–metric comparisons in mean AUROC and AUPRC. Holm–Bonferroni-adjusted advantages reached significance against several baselines, particularly on RPI369 and Aptamer, while the remaining pairwise comparisons consistently retained the same favorable mean direction. Taken together, the complete mean-level leadership across the primary endpoints and the multiple adjusted significant differences identify PRIDA as the best-performing overall method under homology-isolated cross-domain evaluation. Accuracy, Precision, F1, and Sensitivity are secondary threshold-dependent metrics and are reported for completeness.

### 3.3 In-domain interaction prediction

For the in-domain controls, source and target samples were randomly partitioned from the same overall dataset without protein-homology constraints. Every random partition matched its cross-domain counterpart in interaction multiset, source-training size, target-training size, target-test size, and class counts, and identical protein–RNA pairs were kept within one subset. In this protocol, SD-PT was disabled for PRIDA^†^, source-training labels provided supervision, target-training labels were hidden, and CDAN could access target-training samples only as unlabeled data.

As presented in Table 3, the base model without CDAN performed strongly and generally achieved higher AUROC and AUPRC than the CDAN-enhanced version. Because source and target in these controls are random samples from the same overall distribution, they are already closely matched. Using CDAN for distribution alignment on such data may introduce unnecessary noise or incorrect feature maps, messing up already good matching relationships and leading to negative transfer or over-regularization, resulting in performance degradation. Together with the homology-isolated results, this comparison clarifies that CDAN provides its greatest benefit when an explicit source–target shift is present, which is the intended cross-domain setting of PRIDA.

**Table 3:** In-domain performance under random source/target splitting without protein-homology constraints.^a^

Dataset	Models	AUROC	AUPRC	Accuracy	Precision	F1	Sensitivity
RPI488 (random)	PRIDA^†^ (w/CDAN)	0.927±0.028	0.933±0.029	0.873±0.053	0.873±0.122	0.869±0.035	0.884 ± 0.078
	PRIDA^†^ (w/o CDAN)	0.943 ± 0.030	0.955±0.019	0.912±0.028	0.910±0.063	0.906±0.028	**0.905** ± **0.044**
	LPI-BLS	**0.947** ± **0.049**	**0.960** ± **0.034**	**0.941** ± **0.041**	**0.988** ± **0.028**	**0.932** ± **0.049**	0.881±0.069
	SeqMG-RPI	0.917±0.063	0.912±0.086	0.922±0.050	0.956±0.046	0.910±0.060	0.874±0.103
	Transfer-RPI	0.943 ± 0.048	0.958 ± 0.032	0.937 ± 0.037	0.977 ± 0.031	0.927 ± 0.044	0.868±0.063
	CBIL-VHPLI	0.934±0.034	0.944±0.037	0.932±0.032	0.967±0.031	0.922±0.038	0.878±0.059
	RPI-CapsuleGAN	0.931±0.031	0.942±0.031	0.922±0.027	0.946±0.037	0.912±0.032	0.875±0.061
RPI369 (random)	PRIDA^†^ (w/CDAN)	0.740±0.089	0.737±0.110	0.732±0.079	0.701±0.105	0.765 ± 0.050	0.855±0.050
	PRIDA^†^ (w/o CDAN)	**0.785** ± **0.042**	0.750 ± 0.074	**0.759** ± **0.030**	**0.732** ± **0.089**	**0.780** ± **0.016**	0.855±0.104
	LPI-BLS	0.640±0.052*	0.605±0.060*	0.664±0.074	0.628±0.084	0.724±0.038	0.873±0.081
	SeqMG-RPI	0.494±0.076**	0.529±0.048*	0.559±0.055	0.534±0.033	0.691±0.025	**0.982** ± **0.041**
	Transfer-RPI	0.776 ± 0.039	**0.752** ± **0.106**	0.736 ± 0.041	0.716 ± 0.032	0.747±0.047	0.782±0.075
	CBIL-VHPLI	0.521±0.050*	0.501±0.033*	0.536±0.057	0.525±0.043	0.676±0.011	0.964 ± 0.081
	RPI-CapsuleGAN	0.691±0.047	0.706±0.069	0.655±0.071	0.623±0.102	0.724±0.025	0.900±0.122
RPI1807 (random)	PRIDA^†^ (w/CDAN)	0.989 ± 0.005	0.990 ± 0.008	**0.979** ± **0.006**	**0.979** ± **0.011**	**0.980** ± **0.006**	0.982±0.008
	PRIDA^†^ (w/o CDAN)	**0.991** ± **0.002**	**0.993** ± **0.003**	0.971 ± 0.008	0.964±0.012	0.973 ± 0.007	0.983 ± 0.009
	LPI-BLS	0.978±0.015	0.964±0.029	0.964±0.006	0.956±0.009	0.967±0.005	0.978±0.003
	SeqMG-RPI	0.987±0.001	0.988±0.002	0.959±0.009	0.937±0.017	0.963±0.008	**0.992** ± **0.003**
	Transfer-RPI	0.980±0.002*	0.980±0.003	0.968±0.005	0.967 ± 0.010	0.971±0.005	0.975±0.009
	CBIL-VHPLI	0.968±0.009**	0.960±0.019*	0.944±0.013	0.944±0.027	0.948±0.010	0.953±0.013
	RPI-CapsuleGAN	0.974±0.011*	0.960±0.019	0.956±0.010	0.945±0.023	0.960±0.009	0.976±0.029
Aptamer (random)	PRIDA^†^ (w/CDAN)	0.770 ± 0.060	0.770 ± 0.073	0.727±0.098	0.719 ± 0.120	0.758 ± 0.045	0.836±0.126
	PRIDA^†^ (w/o CDAN)	**0.810** ± 0.049*	**0.826** ± **0.061**	**0.739** ± **0.058**	0.711±0.067	**0.761** ± **0.039**	0.824±0.045
	LPI-BLS	0.710±0.068*	0.679±0.060*	0.670±0.089	0.640±0.075	0.722±0.038	0.848±0.103
	SeqMG-RPI	0.534±0.109**	0.510±0.069***	0.624±0.104	0.583±0.073	0.724±0.050	**0.970** ± **0.052**
	Transfer-RPI	0.767±0.022	0.731±0.033	0.733 ± 0.058	**0.765** ± **0.066**	0.741±0.061	0.721±0.078
	CBIL-VHPLI	0.663±0.079**	0.677±0.070*	0.642±0.093	0.629±0.113	0.708±0.025	0.855±0.144
	RPI-CapsuleGAN	0.710±0.068*	0.722±0.067*	0.676±0.085	0.638±0.081	0.731±0.044	0.867 ± 0.035

a Each random partition retained the sample sizes and class counts of the corresponding cross-domain experiment.

† indicates that source-domain supervised pretraining was disabled. Bold and underlined values denote the best and second-best means within each dataset. For AUROC and AUPRC, asterisks indicate Holm–Bonferroni-adjusted significance relative to PRIDA^†^ (w/CDAN).

### 3.4 Ablation study

Table 4 reports the complete ablation study on the homology-isolated partitions. Removing BAN, SD-PT, or CDAN consistently reduced mean AUROC and AUPRC, although the magnitude and statistical strength varied across datasets. The reductions were more evident on RPI369, RPI1807, and Aptamer, whereas the variants remained closer on RPI488.

**Table 4:** Ablation results on the homology-isolated partitions.^a^

Dataset	Models	AUROC	AUPRC	Accuracy	Precision	F1	Sensitivity
RPI488	PRIDA	**0.931** ± **0.016**	**0.941** ± **0.018**	0.907 ± 0.032	0.937 ± 0.066	0.896 ± 0.036	0.863±0.060
	PRIDA (w/o BAN)	0.917±0.042	0.900±0.054	**0.917** ± **0.037**	0.927±0.036	**0.907** ± **0.048**	0.895 ± 0.098
	PRIDA (w/DANN)	0.922 ± 0.030	0.932 ± 0.037	0.907 ± 0.032	**0.947** ± **0.059**	0.894±0.040	0.853±0.078
	PRIDA (w/o CDAN)	0.911±0.026	0.913±0.043	0.888±0.051	0.912±0.116	0.879±0.044	0.863±0.080
	PRIDA (w/o SD-PT)	0.905±0.067	0.901±0.061	0.893±0.064	0.880±0.106	0.888±0.061	**0.905** ± **0.069**
RPI369	PRIDA	**0.597** ± **0.099**	**0.599** ± **0.128**	0.595 ± 0.089	0.572 ± 0.083	0.699 ± 0.028	0.927±0.094
	PRIDA (w/o BAN)	0.409±0.088**	0.469±0.066*	0.541±0.047	0.523±0.026	0.682±0.021	0.982 ± 0.025
	PRIDA (w/DANN)	0.581 ± 0.103	0.591 ± 0.138	**0.623** ± **0.087**	**0.622** ± **0.111**	0.679±0.014	0.791±0.153
	PRIDA (w/o CDAN)	0.437±0.099*	0.460±0.064*	0.550±0.063	0.531±0.043	0.685±0.025	0.973±0.041
	PRIDA (w/o SD-PT)	0.463±0.090*	0.454±0.066*	0.573±0.065	0.541±0.039	**0.700** ± **0.035**	**0.991** ± **0.020**
RPI1807	PRIDA	**0.726** ± **0.081**	**0.738** ± **0.104**	**0.730** ± **0.054**	**0.726** ± **0.118**	**0.779** ± **0.016**	0.875±0.126
	PRIDA (w/o BAN)	0.659±0.164*	0.682±0.160	0.699±0.045	0.664±0.057	0.771±0.021	0.930 ± 0.075
	PRIDA (w/DANN)	0.704 ± 0.088	0.716 ± 0.088	0.718 ± 0.045	0.686 ± 0.053	0.775 ± 0.029	0.900±0.078
	PRIDA (w/o CDAN)	0.653±0.147*	0.674±0.147	0.677±0.085	0.653±0.088	0.760±0.037	**0.931** ± **0.090**
	PRIDA (w/o SD-PT)	0.588±0.060**	0.618±0.055*	0.594±0.073	0.601±0.108	0.712±0.011	0.925±0.150
Aptamer	PRIDA	**0.604** ± **0.033**	**0.662** ± **0.050**	**0.561** ± **0.034**	0.520±0.020	0.681±0.013	0.988 ± 0.027
	PRIDA (w/o BAN)	0.501±0.080*	0.488±0.051***	0.524±0.046	0.514±0.028	0.676±0.016	0.973±0.021
	PRIDA (w/DANN)	0.593 ± 0.031	0.657 ± 0.052	0.539±0.036	0.522 ± 0.020	**0.682** ± **0.015**	0.978±0.036
	PRIDA (w/o CDAN)	0.486±0.030***	0.485±0.037***	0.555 ± 0.059	**0.536** ± **0.045**	0.682 ± 0.013	0.952±0.079
	PRIDA (w/o SD-PT)	0.434±0.081**	0.502±0.074***	0.515±0.019	0.508±0.010	0.674±0.009	**1.000** ± **0.000**

a SD-PT denotes source-domain pretraining, BAN denotes the bilinear attention network, CDAN denotes conditional domain-adversarial learning, and DANN denotes domain-adversarial neural-network alignment. For AUROC and AUPRC, asterisks indicate Holm–Bonferroni-adjusted significance relative to complete PRIDA:

* *P* < 0.05,

** *P* < 0.01, and

*** *P* < 0.001. Bold values indicate the best performance for each metric within each dataset.

Replacing CDAN with unconditional DANN alignment produced comparable results, particularly on RPI369 and Aptamer, but the complete model retained the highest mean AUROC and AUPRC in all four tasks. Together with the random in-domain controls, these results support complementary roles for source-domain representation adaptation, explicit bilinear interaction modeling, and domain alignment, while indicating that the value of adversarial alignment depends on the presence of an explicit source–target shift.

### 3.5 RNA aptamer generation

Figure 2 summarizes the unified evaluation of 10 candidates generated for each of 125 unique Target proteins. For each target protein, each method generates 10 candidate aptamers, grouped by target. The best score is the maximum score within the target-specific candidate set, while the average score is the mean score across the same set.

**Figure 2:**
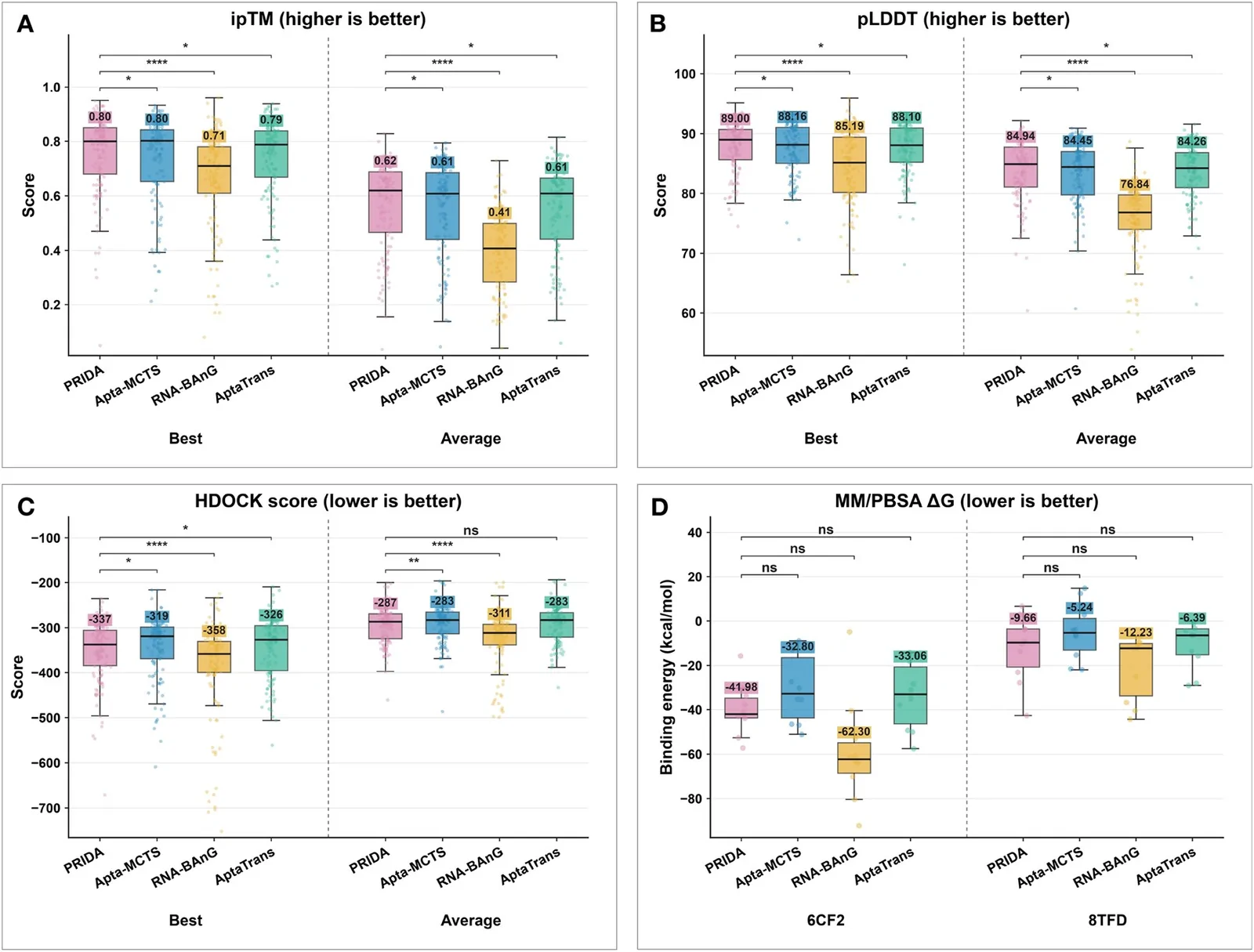
Computational evaluation of generated RNA aptamers. (A) AlphaFold 3 ipTM; (B) AlphaFold 3 pLDDT; (C) HDOCK score; and (D) MM/PBSA-estimated binding free energies for the representative targets 6CF2 and 8TFD. Panels A–C show target-level *Best* and *Average* values across 125 targets. *Best* denotes the maximum ipTM or pLDDT and the minimum HDOCK score. Significance was assessed using target-aligned, two-sided one-sample *t*-tests with Holm–Bonferroni correction. Panel D was analyzed separately for each target using two-sided Welch’s independent-samples *t*-tests with Holm–Bonferroni correction; all comparisons were nonsignificant. Labels indicate means. ${\;}^{*}P<0.05$, ${\;}^{**}P<0.01$, ${\;}^{***}P<0.001$, ${\;}^{****}P<0.0001$; ns, not significant.

PRIDA-guided MCTS achieved the strongest or tied-strongest overall AlphaFold 3 structural confidence under both the Best and Average summaries and remained competitive under HDOCK. It consistently produced more favorable structural-confidence and docking outcomes than the other MCTS-based approaches, Apta-MCTS and AptaTrans, supporting the use of PRIDA as an informative reward model for MCTS-guided sequence search. RNA-BAnG produced the most favorable HDOCK distribution and serves as a strong structure-conditioned reference in the present comparison. An audit based on its published data-construction rules identified potential direct or homologous overlap between 89 of the 125 targets and its training-candidate pool. In addition to the contribution of structural information to aptamer generation, data leakage may also contribute to its improved performance.

For 6CF2 (HIV-1 Rev) and 8TFD (the SARS-CoV-2 nucleocapsid RNA-binding domain), PRIDA yielded more favorable mean MM/PBSA estimates than Apta-MCTS and AptaTrans, whereas RNA-BAnG yielded the lowest values. Although the within-target differences did not reach statistical significance after Holm–Bonferroni correction, these ten-candidate case studies provide complementary preliminary energetic characterization and are interpreted together with the AlphaFold 3 confidence and HDOCK results.

### 3.6 Interpretability

We derived nucleotide-level contribution scores from the bilinear attention weights and averaged them across the five-fold-specific models to explore learned attention patterns. Figure 3 shows the experimentally resolved 5HTO A–C complex (Choi and Ban 2016); minimum nucleotide-to-protein heavy-atom distances and PLIP-identified hydrogen bonds (Adasme *et al.* 2021) provide independent structural annotations. The normalized contribution scores characterize the relative allocation of model attention across nucleotide positions within a sequence, indicating each position’s relative contribution to the prediction. Their formal definition is provided in the Supplementary Material, available as supplementary data at *Bioinformatics* online. The results indicate a certain correlation between the learned attention weights and the interaction regions, especially hydrogen bonds.

**Figure 3:**
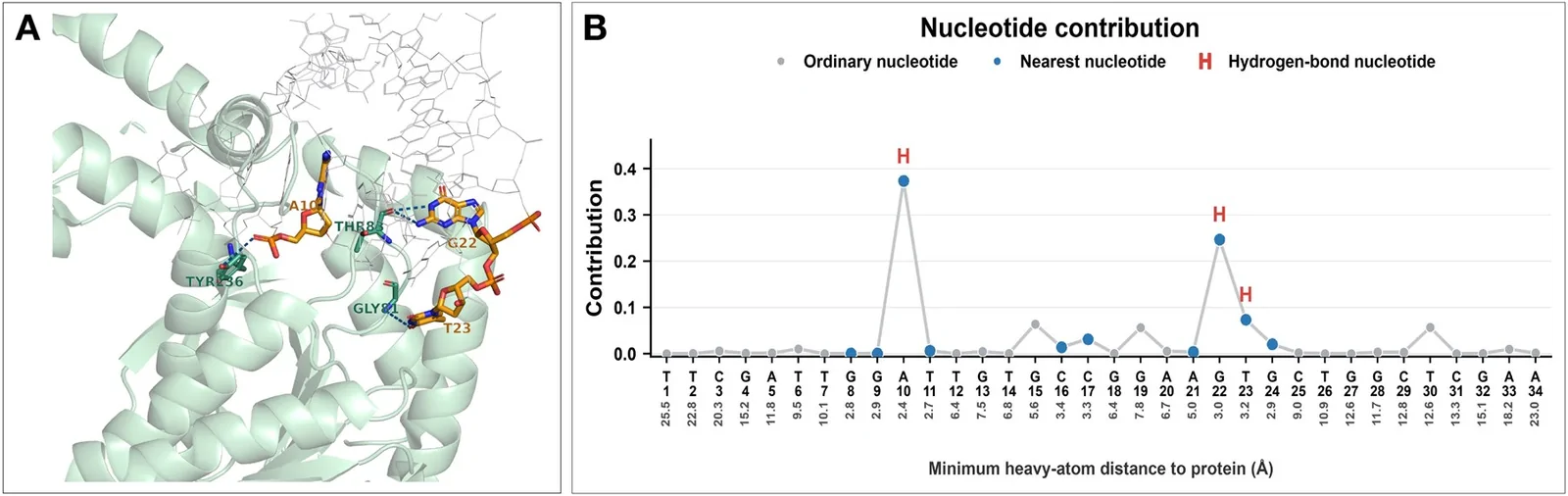
Nucleotide-level interpretability analysis of PRIDA. (A) Experimentally resolved 5HTO protein–nucleic-acid chain complex comprising protein chain A and nucleic-acid chain C. The protein is shown in pale green, the nucleic-acid chain as lines, high-contribution nucleotides as gold sticks, and PLIP-identified hydrogen bonds as cyan dashed lines. (B) Mean nucleotide contribution scores from the five-fold-specific PRIDA models. Grey points denote ordinary nucleotides, blue points denote the 10 nucleotides nearest to the protein, and red H labels denote PLIP-identified hydrogen-bond nucleotides. Values below the positions give the minimum heavy-atom distance to the protein in Å.

To verify this learned pattern, we analyzed 165 experimentally resolved protein–nucleic-acid chain complexes formed by 151 unique protein target chains across 108 PDB entries. For each complex and k∈{1,3,5}, structurally relevant positions were the union of the *k* nearest nucleotides and all hydrogen-bond nucleotides. Enrichment compared the total overlap of attention-ranked Top-*k* positions with this set against the random expected total. The strongest enrichment occurred at Top-3, where attention-ranked positions showed a 1.194-fold enrichment in structurally relevant nucleotides relative to an exact random position (unadjusted one-sided *P* = .00536; Holm–Bonferroni-adjusted *P* = .01609). Top-1 and Top-5 also showed enrichment factors above one (1.152 and 1.024, respectively). A protein-chain-level sensitivity analysis retaining one nucleic-acid-chain partner per protein chain yielded consistent Top-3 enrichment, with adjusted significance in 99.0% of all possible partner selections. The detailed experiment is shown in the Supplementary Material, available as supplementary data at *Bioinformatics* online. These results show that attention contributions may serve as auxiliary evidence for identifying potentially interaction-relevant nucleotide positions.

## 4 Conclusion

In this work, we proposed PRIDA, a conditional domain-adaptive framework for PRI prediction under limited labeled data and protein-level distribution shifts. By integrating pretrained protein and RNA language models with bilinear attention-based interaction modeling and conditional domain-adversarial learning, PRIDA effectively learns transferable interaction representations and improves cross-domain PRI prediction without requiring explicit structural information as input. Coupling the resulting predictor with MCTS further provided useful guidance for computational aptamer search.

Experiments across multiple benchmark datasets show that PRIDA achieves state-of-the-art performance in cross-domain PRI prediction and provides reliable reward signals for aptamer sequence optimization, highlighting its potential to bridge interaction modeling and computational molecular design.

Despite these promising results, several limitations remain. First, PRIDA relies primarily on sequence-derived representations and does not explicitly incorporate high-resolution structural information, which may limit its ability to capture fine-grained atomic interaction mechanisms. Second, negative samples constructed via sequence-based clustering may introduce bias that could affect model robustness in more diverse biological settings. Constructing more reasonable negative samples is an important topic in RNA–protein interaction prediction. Third, the current reinforcement learning-based aptamer generation strategy is still computationally intensive and may require further optimization for large-scale virtual screening.

Future work will focus on integrating structure-aware protein and RNA representations, such as predicted 3D conformations from foundation models, to enhance interaction fidelity. In addition, we will explore more biologically informed negative sampling strategies and experimentally validated benchmark datasets to improve training reliability. Finally, we aim to develop more efficient generative and search strategies to accelerate large-scale aptamer discovery and extend PRIDA to broader biomolecular interaction systems.

## Supplementary Material

btag802_Supplementary_Data
