# Supplementary material for: PRIDA: cross-domain protein–RNA interaction prediction based on biological language models and conditional domain-adversarial learning
Source: Bioinformatics. 2026 Oct 29;42(11):btag802. doi: 10.1093/bioinformatics/btag802 (PMC13767756; doi:10.1093/bioinformatics/btag802)
Supplement: Supplementary file 1 [file btag802_supplementary_data.pdf]

PRIDA: Cross-domain protein-RNA interaction prediction based on biological language models and conditional domain-adversarial learning

Yangyang Zhou†, Jiahao Li†, Simin Liu, Zifan Wang, Runhao Liu,
Zexuan Zhu, Tiantian Zhu*, Jun Zhang*

October 27, 2026

1 Dataset Construction and Homology-Isolated Domain Partitioning

We used RPI369 [1], RPI488 [2], RPI1807 [3], and a protein–aptamer dataset to evaluate PRIDA. The three benchmark datasets were derived from PRIDB [4] or PDB [5] records and curated following the protocol used in RPITER [6]. Protein–aptamer complexes were collected from the PDB using the keyword "aptamer." A resolved protein–aptamer pair was labeled positive when the complex contained at least one intermolecular atom pair below the predefined contact-distance threshold.

Negative Aptamer pairs were constructed by clustering proteins with CD-HIT [7] at a sequence-similarity threshold of 0.4 and pairing a protein with aptamers originating from other protein clusters. This cross-cluster strategy provides biologically informed computational controls while reducing the likelihood of assigning an aptamer to a closely related noncognate target. Because experimentally characterized nonbinding annotations are currently limited, the possibility of latent cross-target binding is considered when interpreting this benchmark.

1.1 Homology Definition and Integrity Audit

Each unique protein sequence was represented as one graph node while all associated protein–RNA interaction records were retained. All protein pairs were compared using MMseqs2 [8] and Needleman–Wunsch global alignment. The latter was implemented with Parasail using BLOSUM62, a gap-opening penalty of 11, and a gap-extension penalty of 1. Complete sequences, including terminal regions, were aligned. Global identity was defined as

$$\text{Global identity} = \frac{N_{\text{identical}}}{L_{\text{global alignment}}} \times 100\%, \tag{S1}$$

where gap columns contributed to $L_{\text{global alignment}}$ but were not counted as identical positions. An edge linked two proteins when either MMseqs2 identity was at least 30% with query and target coverage both at least 80%, or their global identity was at least 30%. Union–Find connected components defined transitive homology groups, which were treated as indivisible assignment units. Post-partition audits confirmed the absence of exact shared sequences, shared homology groups, qualifying MMseqs2 matches, and cross-domain global identities at or above 30%.

1.2 Repeated RPI Source/Target Partitions

For RPI488, RPI369, and RPI1807, a homology group's weight equaled its number of interaction records. Candidate Target sets were generated from homology groups using predefined Target

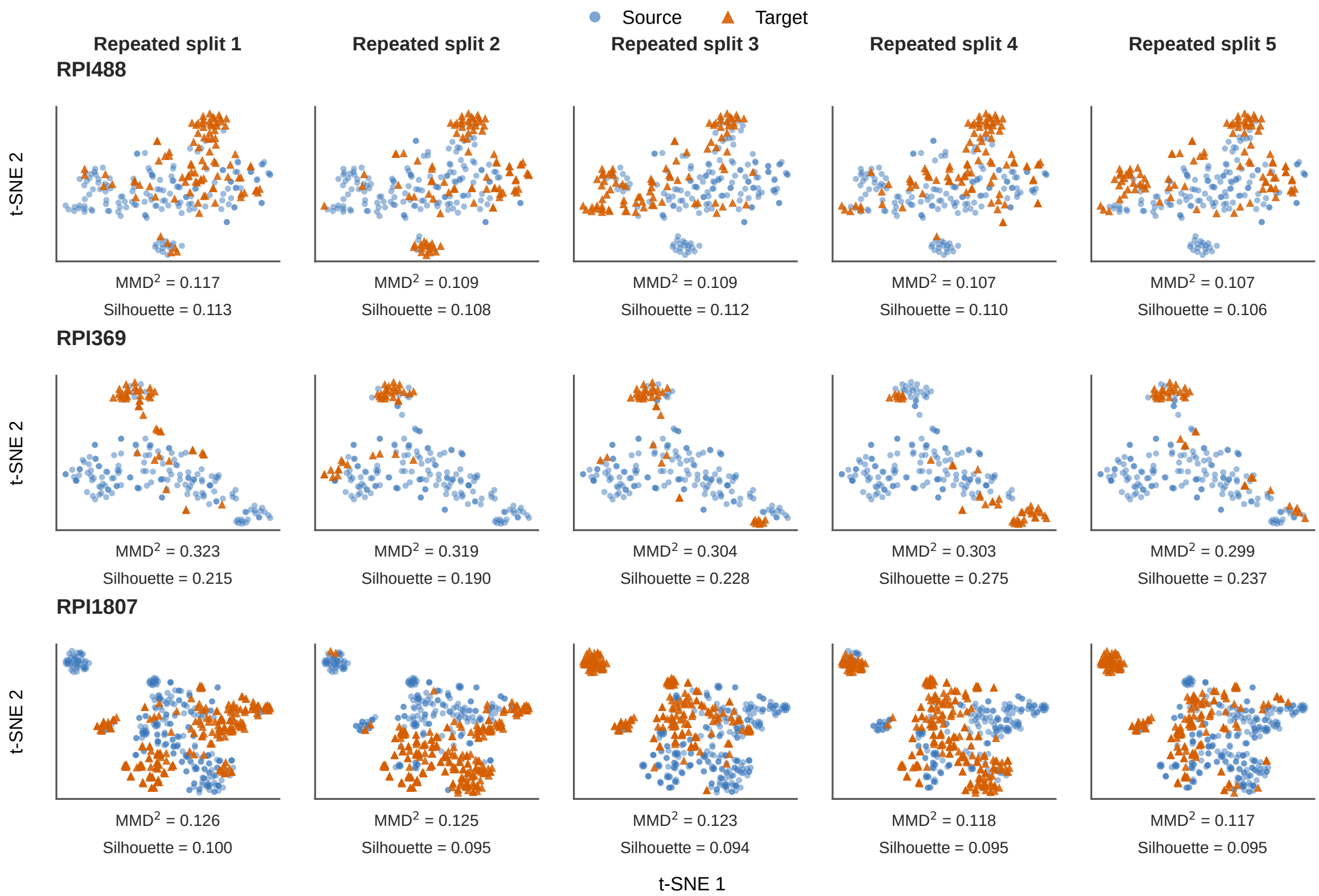

Figure S1: ESM-2-based t-SNE visualization of the five repeated homology-isolated partitions for RPI488, RPI369, and RPI1807. Each point represents one unique protein sequence. The visualization provides an intuitive view of the partition distributions; MMD^2 and Silhouette scores shown in the panels were calculated in the original 1,280-dimensional ESM-2 space, and cross-domain homology isolation was independently verified by the sequence-level audits.

capacities of 203, 220, and 1,537 records, respectively. All remaining groups formed Source, so every interaction record was assigned without deletion and no group was split between domains.

Candidate generation used 30,000 random directions in the 10-dimensional PCA-reduced ESM-2 [9] space. For each direction, groups were ordered by projected score and greedily added, followed by single-group addition or removal to reach the exact Target capacity. Target-training and Target-test records were then assigned by approximately stratified sampling with fixed seeds; repeated protein–RNA keys were indivisible. Homology isolation was required between Source and Target, but not between Target training and Target test because the latter belong to the same domain.

Beam search jointly selected five Target sets per RPI dataset. Pairwise Jaccard similarity between the unique Target protein-sequence sets was constrained to at most 0.40; the observed maximum was 0.380. Figure S1 provides an intuitive visualization of the repeated partitions, and Figure S2 summarizes their overlap. MMD^2 and Silhouette values were calculated in the original 1,280-dimensional ESM-2 space, while cross-domain homology isolation was independently verified through the MMseqs2 and Needleman–Wunsch audits described above.

1.3 Aptamer Source Construction and Target Folds

For Aptamer, domain membership was determined by data origin and the Target domain remained fixed. RPI488, RPI369, and RPI1807 were pooled to broaden protein-family coverage in the Source domain. This design choice was made before model performance was examined and was not based on prediction results. Because RNA length could otherwise provide an unin-

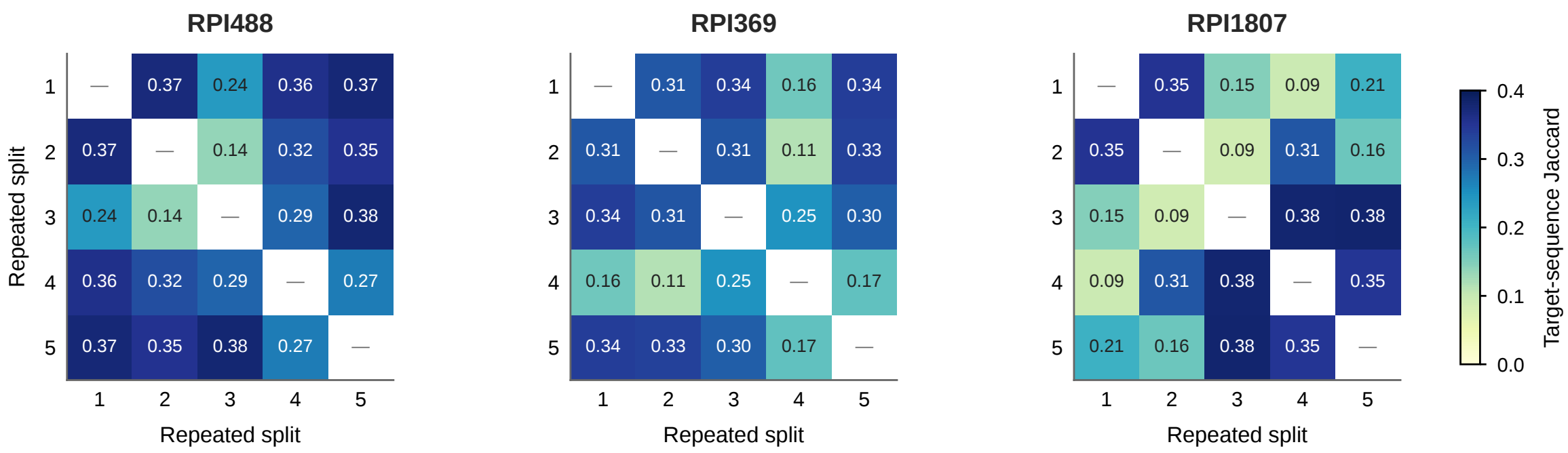

Figure S2: Pairwise Jaccard similarities between Target protein-sequence sets across the five repeated RPI partitions.

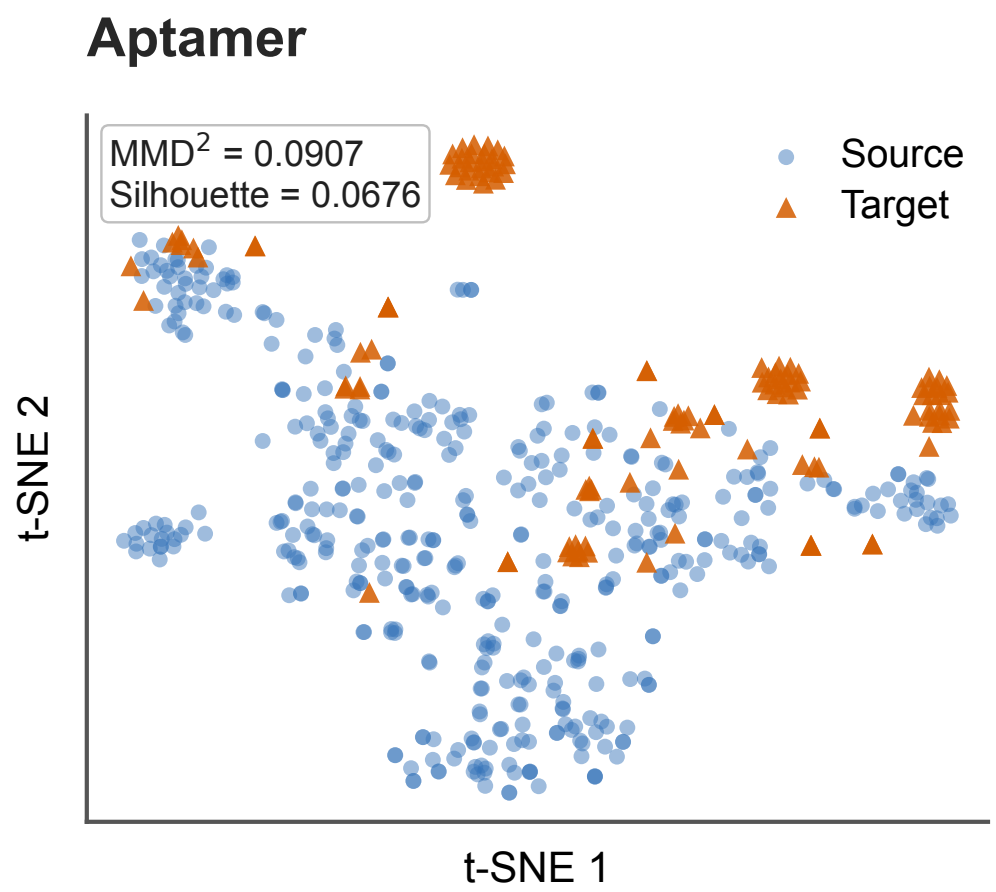

Figure S3: ESM-2-based t-SNE visualization of the fixed Source and Target protein domains in the homology-isolated Aptamer dataset. Each point represents one unique protein sequence. MMD^2 and the Silhouette score were calculated in the original 1,280-dimensional ESM-2 space.

tended domain cue, Source pairs were restricted to RNA length at most 126 nt, the maximum in the Aptamer Target. Proteins connected to any Aptamer Target homology group were then removed using the definition above (Table S1).

The fixed Aptamer Target contained 330 protein–RNA pairs. Stratified five-fold cross-validation produced 264 Target-training and 66 Target-test pairs per fold, with 33 positive and 33 negative test samples. The test folds were mutually exclusive, so every Target pair was evaluated once. In ESM-2 space, the pooled Source and fixed Target had $MMD^2 = 0.0907$ and Silhouette $= 0.0676$ (Figure S3).

1.4 Cross-Domain Sequence-Identity Distribution

Needleman–Wunsch global identity was calculated for every pair of unique Source and Target proteins. For the RPI datasets, each range in Table S2 spans the minimum and maximum of that statistic across the five partitions and is not a confidence interval. Every maximum was below 30%, and no partition contained a cross-domain pair at or above 30%.

Table S1: Changes in interaction-pair counts during construction of the pooled Aptamer Source. Homology removal excludes Source proteins connected to an Aptamer Target homology group.

Dataset	Input pairs	After RNA-length filter	Removed by Target homology	Final Source
RPI488	488	264	1	263
RPI369	738	690	177	513
RPI1807	3237	826	134	692
Total	4463	1780	312	1468

Table S2: Distribution of cross-domain Needleman–Wunsch global sequence identity in the homology-isolated datasets. Ranges for the RPI datasets denote minima and maxima across five partitions.

Dataset	Unique cross-domain protein pairs	Q1 (%)	Median (%)	Q3 (%)	Maximum (%)	Pairs $\geq$ 30%
RPI488	14,062–14,550	11.56–11.86	14.02–14.29	16.07–16.33	29.00	0
RPI369	6,142–6,996	6.59–7.69	9.89–11.39	13.44–14.61	29.20	0
RPI1807	51,704–53,984	8.68–9.05	12.31–12.50	15.24–15.33	29.81	0
Aptamer	52,875	8.28	11.79	14.52	28.03	0

Algorithm S1: Homology-Isolated Protein-Level Domain Partitioning

Input: Protein sequences with interaction-record weights and required Target capacity
Output: Five homology-isolated Source/Target partitions
Compute all-pair MMseqs2 and Needleman–Wunsch similarities;
Connect pairs satisfying either homology criterion and obtain Union–Find components;
Encode unique proteins with ESM-2 and reduce the representations to 10 PCA dimensions;
Generate exact-capacity Target candidates by ordering indivisible components along random projection directions and applying local repair;
Define Source as the complement of each Target candidate;
Retain candidates satisfying class-balance and post-partition homology audits;
Use beam search to select five Target sets with pairwise protein-set Jaccard similarity ≤ 0.40;
Split each Target into approximately stratified training and test records while keeping repeated pair keys indivisible;
return *Five Source-training, Target-training, and Target-test assignments*

1.5 Details of MCTS-based Aptamer Sequence Generation

We implemented Monte Carlo Tree Search (MCTS) [10] to generate target-oriented RNA sequences autoregressively. Each node represents a partial sequence in a two-character encoding; each action appends one nucleotide with a directional marker, yielding eight expansion states. The root is empty, and expansion continues to a fixed length of 20 nt. This common length follows recent 20-nt aptamer-generation precedent [11] and provides a uniform, reproducible, length-matched evaluation setting for all methods.

Every iteration comprises selection, expansion, simulation, and backpropagation. Selection uses the Upper Confidence Bound for Trees criterion to balance accumulated rewards and visit counts. During simulation, random actions complete the current partial sequence; the resulting RNA string is encoded and combined with the fixed target-protein representation. For PRIDA-guided MCTS, the reward for a completed candidate is the arithmetic mean of its positive-interaction probabilities from the five fold-specific models trained on the homology-isolated

Aptamer task. The reward is propagated through the selected path, and the highest-UCT subsequence becomes the fixed prefix for the next search round. No ground-truth Target label is accessed during generation.

1.6 Aptamer Generation and Computational Evaluation

The 151 PDB-chain records in the Aptamer Target represented 125 unique protein sequences; identical sequences were evaluated once to avoid repeated target weighting. For each target, PRIDA, Apta-MCTS [10], AptaTrans [12], and RNA-BAnG [13] each generated ten 20-nt RNAs, yielding 1,250 candidates per method. PRIDA, Apta-MCTS, and AptaTrans use MCTS with different reward models, whereas RNA-BAnG is a structure-conditioned reference.

AlphaFold 3 [14] provided ipTM and pLDDT values, and HDOCK [15] provided an independent docking score. For every target and method, *Best* was the maximum ipTM or pLDDT and the minimum HDOCK score among ten candidates; *Average* was the arithmetic mean. Average and Best were compared only within the same metric–summary family. Target-aligned, two-sided one-sample t-tests were applied to paired PRIDA–baseline differences across 125 targets, and the three p-values within each of the six metric–summary families were corrected by Holm–Bonferroni.

Two targets were selected for molecular-dynamics evaluation because of their distinct biological relevance: 6CF2 is HIV-1 Rev, whose cognate RNA interaction is essential to the viral life cycle, and 8TFD is the RNA-binding domain of the SARS-CoV-2 nucleocapsid protein in an aptamer complex. All ten candidates per method and target were simulated for 50 ns in GROMACS [16] with the CHARMM36 force field [17], followed by MM/PBSA estimation of ΔG_{bind} [18]. Because sequences from different methods had no one-to-one correspondence, within-target comparisons used two-sided Welch independent-samples t-tests and Holm–Bonferroni correction.

We additionally reconstructed the candidate training pool implied by the published RNA-BAnG data rules. Potential direct or homologous overlap was found for 89 of 125 targets: 81 had a same-PDB-chain record, seven had a high-coverage homolog from another entry, and one had uncertain exact-entry timing but an earlier high-coverage homolog. Because the final training manifest was unavailable, this audit context was incorporated into result interpretation. RNA-BAnG therefore serves as a strong structure-conditioned reference in the present comparison, including the 6CF2 and 8TFD cases.

1.7 Attention-Based Nucleotide Contribution Analysis

Let $A_{hij}^{(f)}$ denote the attention score from head h of fold-specific model f between nucleotide i and protein position j. Scores were first averaged across H heads:

$$S_{ij}^{(f)} = \frac{1}{H}\sum_{h=1}^{H} A_{hij}^{(f)}. \tag{S2}$$

They were then softmax-normalized across nucleic-acid positions for each protein position and averaged over protein positions and the five models:

$$c_i = \frac{1}{5L_P}\sum_{f=1}^{5}\sum_{j=1}^{L_P}\frac{\exp\left(S_{ij}^{(f)}\right)}{\sum_{u=1}^{L_N}\exp\left(S_{uj}^{(f)}\right)}, \qquad \sum_{i=1}^{L_N} c_i = 1, \tag{S3}$$

where L_N and L_P are the represented nucleic-acid and protein lengths. Thus, c_i characterizes the relative allocation of model attention across nucleotide positions and summarizes each position's relative contribution to the prediction, supporting comparison of potentially structure-relevant regions within a sequence.

The representative 5HTO A–C complex [19] was annotated with each nucleotide's minimum heavy-atom distance to the protein and PLIP-identified hydrogen bonds [20]. Cohort-level analysis used 165 experimentally resolved protein–nucleic-acid chain complexes formed by pairing 151 unique protein target chains with their interacting nucleic-acid-chain partners across 108 PDB entries. Twelve protein chains had more than one nucleic-acid-chain partner, producing 14 additional chain-level combinations and giving $151 + 14 = 165$ complexes. For each complex and $k \in \{1, 3, 5\}$, structurally relevant positions were the union of the k nearest nucleotides and all hydrogen-bond nucleotides. Enrichment compared the total overlap of attention-ranked Top-k positions with this set against the random expected total. The exact null distribution was obtained by convolving complex-specific hypergeometric probability mass functions; the three one-sided exact p-values were Holm–Bonferroni corrected.

To verify that the enrichment was robust to the choice of nucleic-acid-chain partner, we performed a protein-chain-level sensitivity analysis using the 151 unique protein target chains. Among these chains, 139 had one nucleic-acid-chain partner and therefore contributed the same fixed complex to every sensitivity dataset. Of the remaining 12 chains, ten each had two nucleic-acid-chain partners, whereas two (8g8z_I and 8g8z_J) each had three. Selecting one nucleic-acid-chain partner for each of these 12 chains yielded $2^{10} \times 3^2 = 9{,}216$ possible selections. Combining each selection with the 139 fixed complexes produced 9,216 alternative datasets, each containing one protein–nucleic-acid chain complex for every protein target chain, or 151 complexes in total. The exact random-position enrichment test and Holm–Bonferroni correction across Top-1, Top-3, and Top-5 were repeated for every dataset, directly evaluating robustness to the selection of nucleic-acid-chain partners. Complexes originating from the same PDB entry may retain shared structural context.

In the primary analysis of protein–nucleic-acid chain complexes, the strongest enrichment signal occurred at Top-3, with an enrichment factor of 1.194 (unadjusted $p = 0.00536$, adjusted $p = 0.01609$). Top-1 and Top-5 also yielded enrichment factors above one (1.152 and 1.024, respectively), although their adjusted tests did not reach significance (unadjusted $p = 0.171$ and 0.315; both adjusted $p = 0.342$). Across the 9,216 alternative datasets, Top-3 enrichment ranged from 1.162 to 1.240 and retained Holm–Bonferroni-adjusted significance in 9,128 datasets (99.0%); the remaining 88 datasets did not reach adjusted significance. This consistency demonstrates strong robustness to the selection of nucleic-acid-chain partners. Top-1 and Top-5 remained non-significant throughout. Together, these results support attention scores as auxiliary evidence for identifying potentially interaction-relevant nucleotide positions, with complementary structural or experimental evidence able to further strengthen site-level interpretation.

1.8 Evaluation Metrics

For RPI prediction, we primarily evaluate classification performance using the area under the receiver operating characteristic curve (AUROC) and the area under the precision–recall curve (AUPRC). We additionally report Accuracy, Precision, F1 score, and Sensitivity. For threshold-dependent metrics, prediction scores are binarized using the decision threshold that maximizes the F1 score. All metrics are reported with three decimal places, and higher values indicate better performance.

Let TP, TN, FP, and FN denote the numbers of true positives, true negatives, false positives, and false negatives, respectively. Accuracy measures the proportion of correctly classified samples:

$$\text{Accuracy} = \frac{TP + TN}{TP + TN + FP + FN}. \tag{S4}$$

Precision measures the proportion of predicted positive samples that are truly positive:

$$\text{Precision} = \frac{TP}{TP + FP}. \tag{S5}$$

Sensitivity, also known as recall or true positive rate (TPR), measures the proportion of positive samples that are correctly identified:

$$\text{Sensitivity} = \text{Recall} = \frac{TP}{TP + FN}. \tag{S6}$$

The F1 score is the harmonic mean of Precision and Recall:

$$\text{F1} = \frac{2 \times \text{Precision} \times \text{Recall}}{\text{Precision} + \text{Recall}}. \tag{S7}$$

AUROC evaluates the ranking ability of the model by measuring the area under the ROC curve, which plots the true positive rate against the false positive rate (FPR) at different thresholds:

$$\text{AUROC} = \int_0^1 \text{TPR}(\text{FPR})\, d\text{FPR}, \tag{S8}$$

where

$$\text{TPR} = \frac{TP}{TP + FN}, \quad \text{FPR} = \frac{FP}{FP + TN}. \tag{S9}$$

AUPRC measures the area under the precision–recall curve and is particularly informative when the class distribution is imbalanced:

$$\text{AUPRC} = \int_0^1 \text{Precision}(\text{Recall})\, d\text{Recall}. \tag{S10}$$

Interaction-prediction results are reported as the mean $\pm$ sample standard deviation over five matched Source/Target partitions for each RPI dataset or five Target folds for Aptamer. AUROC and AUPRC were designated as primary endpoints. For every PRIDA–comparator contrast, a two-sided one-sample t-test was applied to the five fold-aligned performance differences ($df = 4$). Within each dataset, AUROC and AUPRC p-values were adjusted using the Holm–Bonferroni procedure. Adjusted thresholds are denoted by $^{*}p < 0.05$, $^{**}p < 0.01$, and $^{***}p < 0.001$. Accuracy, Precision, F1, and Sensitivity are reported as secondary descriptive metrics without significance annotations.

References

[1] Usha K Muppirala, Vasant G Honavar, and Drena Dobbs. Predicting rna-protein interactions using only sequence information. *BMC Bioinformatics*, 12(1):489, 2011.

[2] Xiaoyong Pan, Yong-Xian Fan, Junchi Yan, and Hong-Bin Shen. Ipminer: hidden ncrna-protein interaction sequential pattern mining with stacked autoencoder for accurate computational prediction. *BMC Genomics*, 17(1):582, 2016.

[3] V Suresh, Liang Liu, Donald Adjeroh, and Xiaobo Zhou. Rpi-pred: predicting ncrna-protein interaction using sequence and structural information. *Nucleic Acids Research*, 43(3):1370–1379, 2015.

[4] Benjamin A Lewis, Rasna R Walia, Michael Terribilini, Jeff Ferguson, Charles Zheng, Vasant Honavar, and Drena Dobbs. Pridb: a protein–rna interface database. *Nucleic Acids Research*, 39(suppl_1):D277–D282, 2010.

[5] Helen M Berman, John Westbrook, Zukang Feng, Gary Gilliland, Talapady N Bhat, Helge Weissig, Ilya N Shindyalov, and Philip E Bourne. The protein data bank. *Nucleic Acids Research*, 28(1):235–242, 2000.

[6] Cheng Peng, Siyu Han, Hui Zhang, and Ying Li. Rpiter: a hierarchical deep learning framework for ncrna–protein interaction prediction. *International Journal of Molecular Sciences*, 20(5):1070, 2019.

[7] Limin Fu, Beifang Niu, Zhengwei Zhu, Sitao Wu, and Weizhong Li. Cd-hit: accelerated for clustering the next-generation sequencing data. *Bioinformatics*, 28(23):3150–3152, 2012.

[8] Martin Steinegger and Johannes Söding. MMseqs2 enables sensitive protein sequence searching for the analysis of massive data sets. *Nature Biotechnology*, 35(11):1026–1028, 2017.

[9] Zeming Lin, Halil Akin, Roshan Rao, Brian Hie, Zhongkai Zhu, Wenting Lu, Nikita Smetanin, Robert Verkuil, Ori Kabeli, Yaniv Shmueli, et al. Evolutionary-scale prediction of atomic-level protein structure with a language model. *Science*, 379(6637):1123–1130, 2023.

[10] Gwangho Lee, Gun Hyuk Jang, Ho Young Kang, and Giltae Song. Predicting aptamer sequences that interact with target proteins using an aptamer-protein interaction classifier and a monte carlo tree search approach. *PLOS ONE*, 16(6):e0253760, 2021.

[11] Jun Zhang, Ju Zhang, Shaoxuan Tang, Chuancheng Liu, Yushan Cai, Hao Zeng, Xiangjie Meng, Bei Liu, Yang Zhang, and Yu Wang. Single-round evolution of rna aptamers with grape-lm. *Nature Biotechnology*, 2026.

[12] Incheol Shin, Keumseok Kang, Juseong Kim, Sanghun Sel, Jeonghoon Choi, Jae-Wook Lee, Ho Young Kang, and Giltae Song. Aptatrans: a deep neural network for predicting aptamer-protein interaction using pretrained encoders. *BMC Bioinformatics*, 24(1):447, 2023.

[13] Roman Klypa, Alberto Bietti, and Sergei Grudinin. Bang: Bidirectional anchored generation for conditional rna design. *arXiv preprint arXiv:2502.21274*, 2025.

[14] Josh Abramson, Jonas Adler, Jack Dunger, Richard Evans, Tim Green, Alexander Pritzel, Olaf Ronneberger, Lindsay Willmore, Andrew J Ballard, Joshua Bambrick, et al. Accurate structure prediction of biomolecular interactions with alphafold 3. *Nature*, 630(8016):493–500, 2024.

[15] Yumeng Yan, Di Zhang, Pei Zhou, Botong Li, and Sheng-You Huang. HDOCK: a web server for protein–protein and protein–dna/rna docking based on a hybrid strategy. *Nucleic Acids Research*, 45(W1):W365–W373, 2017.

[16] Mark James Abraham, Teemu Murtola, Roland Schulz, Szilárd Páll, Jeremy C. Smith, Berk Hess, and Erik Lindahl. GROMACS: High performance molecular simulations through multi-level parallelism from laptops to supercomputers. *SoftwareX*, 1–2:19–25, 2015.

[17] Elizabeth J. Denning, U. Deva Priyakumar, Lennart Nilsson, and Alexander D. MacKerell. Impact of 2′-hydroxyl sampling on the conformational properties of RNA: Update of the CHARMM all-atom additive force field for RNA. *Journal of Computational Chemistry*, 32(9):1929–1943, 2011.

[18] Bill R. Miller, T. Dwight McGee, Jason M. Swails, Nadine Homeyer, Holger Gohlke, and Adrian E. Roitberg. MMPBSA.py: An efficient program for end-state free energy calculations. *Journal of Chemical Theory and Computation*, 8(9):3314–3321, 2012.

[19] Sung-Jin Choi and Changill Ban. Crystal structure of a DNA aptamer bound to PvLDH elucidates novel single-stranded DNA structural elements for folding and recognition. *Scientific Reports*, 6(1):34998, 2016.

[20] Melissa F. Adasme, Katja L. Linnemann, Sarah Naomi Bolz, Florian Kaiser, Sebastian Salentin, V. Joachim Haupt, and Michael Schroeder. PLIP 2021: expanding the scope of the protein–ligand interaction profiler to DNA and RNA. *Nucleic Acids Research*, 49(W1):W530–W534, 2021.
